# Microsecond-long simulation reveals the molecular mechanism for the dual inhibition of falcipain-2 and falcipain-3 by antimalarial lead compounds

**DOI:** 10.3389/fmolb.2022.1070080

**Published:** 2022-12-19

**Authors:** Ammar Usman Danazumi, Emmanuel Oluwadare Balogun

**Affiliations:** ^1^ Faculty of Chemistry, Warsaw University of Technology, Warsaw, Poland; ^2^ Groningen Research Institute of Pharmacy, University of Groningen, Groningen, Netherlands; ^3^ Department of Biochemistry, Ahmadu Bello University, Zaria, Nigeria; ^4^ Africa Centre of Excellence for Neglected Tropical Diseases and Forensic Biotechnology, Ahmadu Bello University, Zaria, Nigeria; ^5^ Center for Discovery and Innovation in Parasitic Diseases, Skaggs School of Pharmacy and Pharmaceutical Sciences, University of California, San Diego, San Diego, CA, United States; ^6^ Department of Biomedical Chemistry, Graduate School of Medicine, The University of Tokyo, Tokyo, Japan

**Keywords:** malaria, *Plasmodium falciparum*, falcipains, molecular dynamics simulation, enzyme inhibition

## Abstract

The latest world malaria report revealed that human deaths caused by malaria are currently on the rise and presently stood at over 627,000 per year. In addition, more than 240 million people have the infection at any given time. These figures make malaria the topmost infectious disease and reiterate the need for continuous efforts for the development of novel chemotherapies. Malaria is an infectious disease caused majorly by the protozoan intracellular parasite *Plasmodium falciparum* and transmitted by mosquitoes. Reports abound on the central role of falcipains (cysteine protease enzymes) in the catabolism of hemoglobin for furnishing the plasmodium cells with amino acids that they require for development and survival in the hosts. Even though falcipains (FPs) have been validated as drug target molecules for the development of new antimalarial drugs, none of its inhibitory compounds have advanced beyond the early discovery stage. Therefore, there are renewed efforts to expand the collection of falcipain inhibitors. As a result, an interesting finding reported the discovery of a quinolinyl oxamide derivative (QOD) and an indole carboxamide derivative (ICD), with each compound demonstrating good potencies against the two essential FP subtypes 2 (FP-2) and 3 (FP-3). In this study, we utilized microsecond-scale molecular dynamics simulation computational method to investigate the interactions between FP-2 and FP-3 with the quinolinyl oxamide derivative and indole carboxamide derivative. The results revealed that quinolinyl oxamide derivative and indole carboxamide derivative bound tightly at the active site of both enzymes. Interestingly, despite belonging to different chemical scaffolds, they are coordinated by almost identical amino acid residues *via* extensive hydrogen bond interactions in both FP-2 and FP-3. Our report provided molecular insights into the interactions between FP-2 and FP-3 with quinolinyl oxamide derivative and indole carboxamide derivative, which we hope will pave the way towards the design of more potent and druglike inhibitors of these enzymes and will pave the way for their development to new antimalarial drugs.

## 1 Introduction

Malaria continues to pose significant public health threats in the tropical and subtropical regions, given a global estimate of about 241 million cases in 2020, with yearly deaths reaching a record high of 627,000 in the same year. Malaria is an infectious disease caused by multiple species of intra-erythrocytic parasites belonging to the genus *Plasmodium*. The five species that are responsible for the disease in humans are *Plasmodium falciparum*, *P. vivax*, *P. malariae*, *P. ovale*, and *P. knowlesi*. From the standpoint of global health, *P. falciparum* is the most important because it is responsible for over 90% of global malaria cases and mortalities (Snow, 2015). The current situation is aggravated by the increased resistance to insecticides by the mosquito vectors as well as the spread of drug resistance to the available antimalarial drugs (WHO, 2022). This justified the unrelenting efforts towards the design and development of new drug candidates against malaria, along with expanding the plasmodial drug targets pool. Cysteine proteases have been identified as molecular targets that have become attractive for drug design not only against *Plasmodium sp* but also in related parasitic diseases (McKerrow et al., 1999; Pandey and Dixit, 2012; Siqueira-Neto et al., 2018).

**FIGURE 1:**
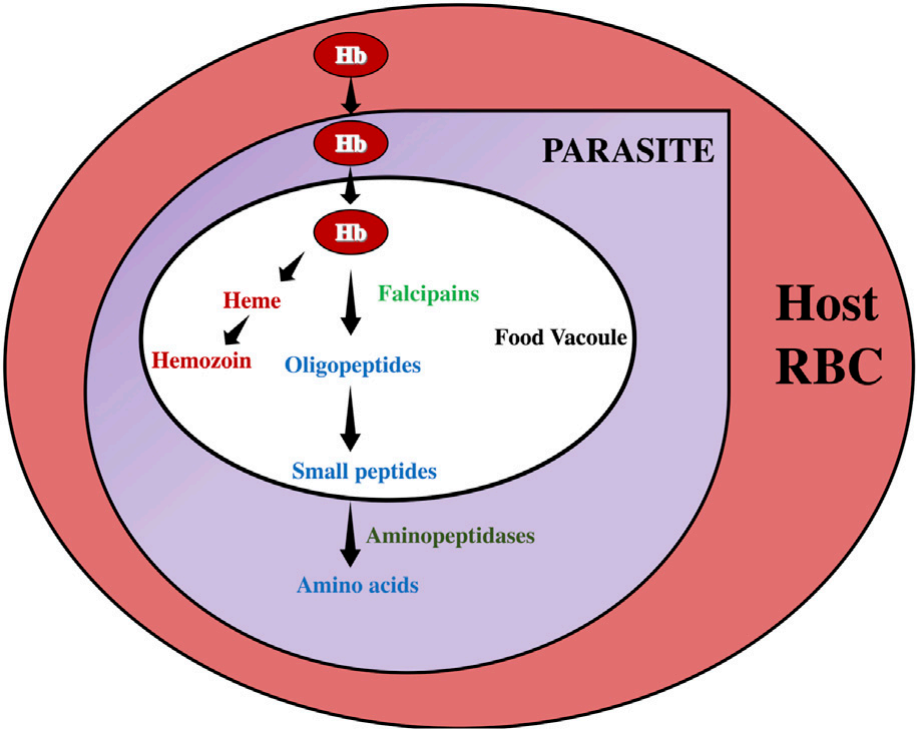
Simplified representation of hemoglobin catabolism in *Plasmodium falciparum*. Hemoglobin is denoted by Hb.

Falcipains (FPs) are important cysteine proteases of *P. falciparum* that are central to acquisition by the parasite. To ensure the survival of Plasmodium in mammalian hosts, FPs catalyze the digestion of host hemoglobin in the food vacuole of Plasmodium to maintain the amino acid supply to the parasite (Hanspal et al., 2002). Figure 1 summarizes the pathway of hemoglobin catabolism in *P. falciparum*, highlighting the role of falcipains in ensuring amino acids supply for growth and development. There are two subfamilies of FPs, FP-1, and FP-2/3. While FP-1 is not relevant to the intra-erythrocytic human stage of the parasite, FP-2/3 are essential as gene deletions of FP2/3 are lethal to *P. falciparum* (Rosenthal, 2020). FPs have been genetically characterized, with FP-2 and FP-3 sharing 68% identity, and happened to be critical for the erythrocytic stage of the parasite’s life cycle in the host (Marco and Miguel Coteron, 2012). Although FP-2 is the chief haemoglobinase of *Plasmodium falciparum* (Hanspal et al., 2002), concomitant inhibition of FP-2 and FP-3 is necessary to cut-off the parasite’s amino acids supply and thus becomes an effective therapeutic target against *P falciparum* (Ettari et al., 2021).

**FIGURE 2:**
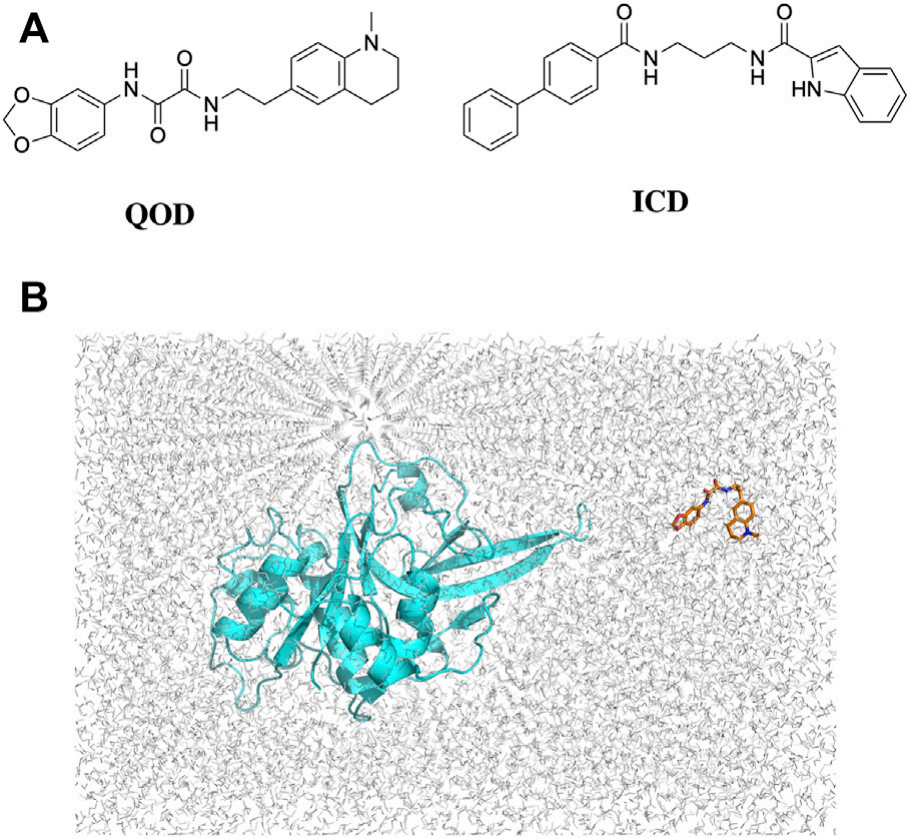
2D structures of QOD and ICD **(A)**. Position of falcipain-2 and *N*-(2H-1,3-benzodioxol-5-yl)-*N'*-[2-(1-methyl-1,2,3,4-tetrahydroquinolin-6-yl)ethyl]ethanediamide (QOD) in a cubic simulation box **(B)**.

Several inhibitors of these targets have been reported, but none have yet reached clinical trials (Pant et al., 2018; Machin et al., 2019; Hernandez Gonzalez et al., 2022). Among others, a quinolinyl oxamide derivative (QOD), *N*-(2H-1,3-benzodioxol-5-yl)-*N'*-[2-(1-methyl-1,2,3,4-tetrahydroquinolin-6-yl) ethyl]ethanediamide and an indole carboxamide derivative (ICD), *N-*{3-[(biphenyl-4-yl carbonyl) amino]propyl}-1H-indole-2-carboxamide (herein referred to as compound QOD and ICD, respectively Figure 2A) were reported as potent dual inhibitors of FP-2 and FP-3 (Rana et al., 2020). However, the molecular/structural explanation for their dual inhibitory activities has not been established. However, there is a knowledge gap in the mechanisms of the dual inhibitory nature of the QOD and ICD due to lack of structural data, which has limited SAR studies for obtaining more efficient and more potent inhibitory derivatives. Further development of these compounds into active and non-toxic drugs depends heavily on deciphering the mechanism of their interaction with their molecular targets. This information is often harnessed from Biophysical techniques, including nuclear magnetic resonance spectroscopy, X-ray crystallography, or even cryo-electron microscopy (Batool et al., 2019). Although these techniques remain the gold standards for structure-based drug design, information on time-dependent dynamic interaction is difficult–and in some cases impossible to derive from such approaches. On the contrary, molecular dynamics (MD) simulations have emerged as a versatile computational method that offers time-resolved dynamic behavior of biomolecules and have become an established method for studying the dynamics of protein-ligand interactions (Hollingsworth and Dror, 2018). Consequently, we used such an approach to unravel the mechanism of dual inhibition of FP-2 and FP-3 by antimalarial lead compounds QOD and ICD (Rana et al., 2020). To mimic the natural ligand binding, we placed the compounds away from the proteins and allowed them to diffuse freely in the simulation box to find their preferred binding site. By subjecting our systems to microsecond-long MD simulations, we gained atomic-scale insight into the binding mechanism of the compounds QOD and ICD to both FP-2 and FP-3. In addition to providing the molecular basis for the dual inhibitory activities of the compounds to these essential FPs, our findings will accelerate the optimization of QOD and ICD towards the development of new classes of antimalarials.

## 2 Methods

### 2.1 Structure preparation

The crystal structures of FP-2 and FP-3 were obtained from the protein databank (PDB) with the IDs 3BPF and 3BPM, respectively. Co-crystallized water molecules and other heteroatoms were removed, and Hydrogen atoms were added to the structures using the H++ server (Gordon et al., 2005). The 3D coordinates of the compounds QOD and ICD were collected from the MolPort database in SDF format, protonated, and converted to PDB format using the Schrodinger Maestro suite (Schrödinger, 2022). The cleaned-protonated proteins and ligands structures were then used to generate topology in the amber forcefield using AmberTools22 (Case et al., 2017). In each case, the ligand (i.e., QOD or ICD) is placed in a random position away from the protein (FP-2 or FP-3) in a cubic simulation box (Figure 2B) and solvated in the TIP3P water model (Price and Brooks, 2004). The systems were further neutralized with 150 mM NaCl, and ligand parameters were defined using GAFF forcefield (Wang et al., 2004).

### 2.2 Molecular dynamics simulations

All MD simulations were performed using GROMACS-2021.3 (Berendsen et al., 1995). Accordingly, each system was energy minimized for 5000 steps with the steepest descent algorithm followed by equilibration in the NVT ensemble for 100 ps to a temperature of 298 K using the velocity rescale temperature coupling (Bussi et al., 2007). The systems were then equilibrated in an NPT ensemble for 2 ns to a pressure of 1 atmosphere, using the Parrinello-Rahman barostat for pressure coupling (Parrinello and Rahman, 1998). This was followed by 1 𝛍s long final production mdrun in NPT ensemble, saving snapshots after every 10 ps. The complete simulation input parameters are described in the Supplementary Material.

### 2.3 MD simulation data analyses

To establish the stability of the systems, the trajectories generated from the 1 𝛍s simulations were used to calculate the root-mean-square deviation (RMSD) of the protein’s backbone atoms. Unless told otherwise, the last 500 ns of the trajectories were used for other downstream analyses, such as the root-mean-square fluctuations (RMSF). Clustering analysis was performed to classify the different binding orientations of the ligands. The clustering was done using the GROMACS gmx cluster module, and the GROMOS method was chosen as the classification method (Daura et al., 1998). Clusters are separated by a 0.25 nm difference in RMSD. The calculation was performed from a snapshot every 10 ps.

### 2.4 Molecular mechanics generalized born and surface area continuum solvation (MM/GBSA) binding free energy calculation

The binding energies of the interaction between QOD, ICD, and FP-2 or FP-3 were calculated from the last 500 ns of the MD simulation trajectories using the molecular mechanics generalized Born and surface area continuum solvation approach. In this method, the binding free energy is given by:

$$\Delta G_{bind}=\Delta H-T\Delta S⋍\Delta E_{MM}+\Delta G_{sol}-T\Delta S$$ (1)

$$\Delta E_{MM}=\Delta E_{\mathrm{int}ernal}+\Delta E_{electrostatic}+\Delta E_{vdw}$$ (2)

$$\Delta G_{sol}=\Delta G_{GB}+\Delta G_{SA}$$ (3)

(Hou et al., 2011)

The term ΔE_MM_ represents energy due to change in the gas phase, which is defined by internal (bond, angle, and dihedral energies, ΔE_internal_), electrostatic (ΔE_electrostatic_), and van der Waals energies (ΔE_vdw_), while ΔG_sol_ and TΔS account for the free energy of solvation and entropy due to binding-induced conformational changes, respectively. The solvation free energy is a term that is derived from the sum of electrostatic (ΔG_GB_, estimated from the Generalised Born model) and non-electrostatic (ΔG_SA_) solvation energy components, which is calculated using the solvent accessible surface area (Genheden and Ryde, 2015). The binding free energy calculation was performed using gmx_MMPBSA software, and the input parameters are described in the Supplementary Material (Valdés-Tresanco et al., 2021).

## 3 Results

### 3.1 Assessing the stability of the systems

**FIGURE 3:**
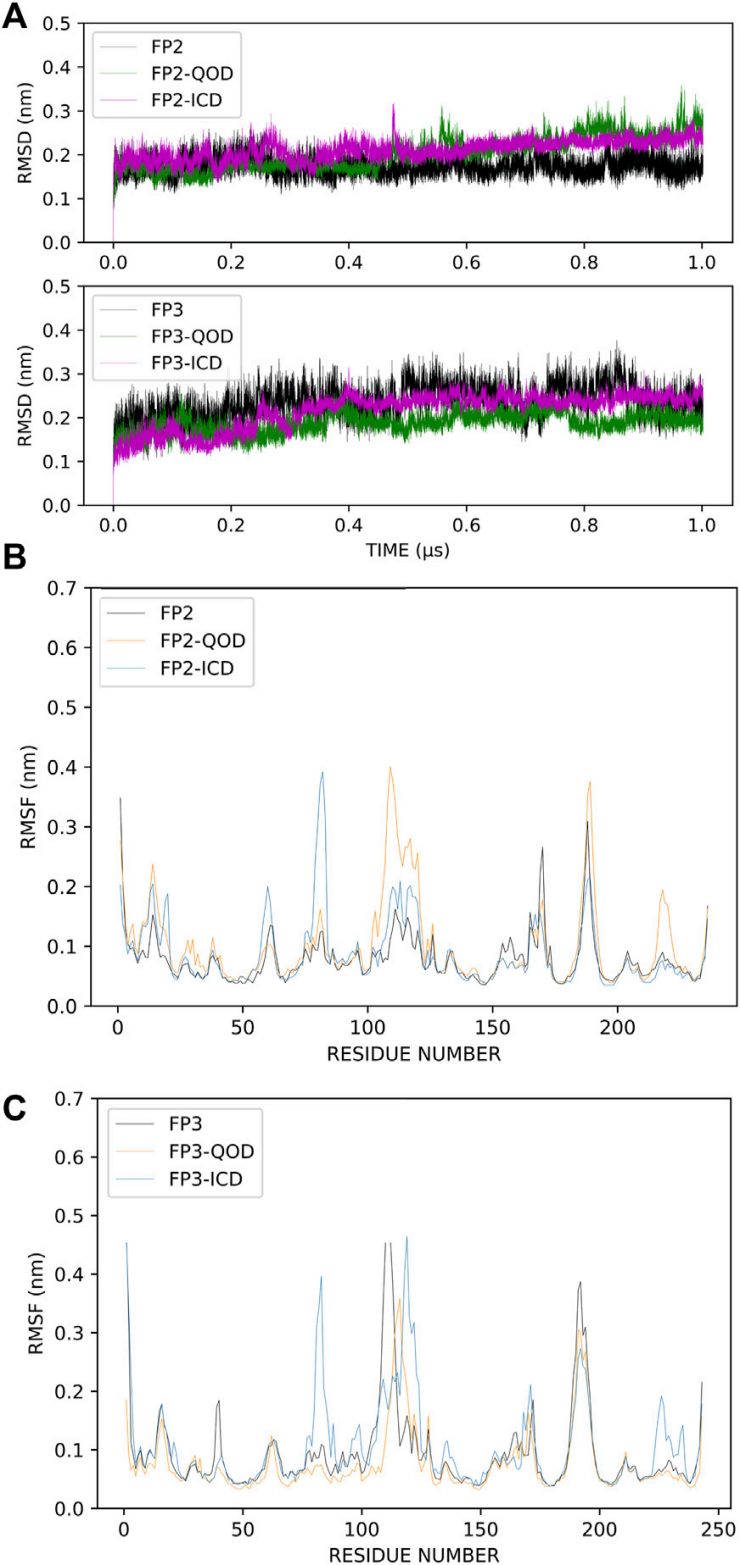
Root-mean-square deviations (RMSD) of the protein backbone atoms of the different simulation systems. Apo-FP2/FP3, FP2/FP3-QOD, FP2/FP3-ICD are represented in black, green and magenta lines, respectively. **(A)**. Root-mean-square fluctuations (RMSF) of C-α atoms of FP-2 simulation systems. Apo-FP2, FP2-QOD, FP2-ICD are represented in black, orange and light blue lines, respectively. **(B)**. Root-mean-square fluctuations (RMSF) of C-α atoms of FP-3 simulation systems. Apo-FP3, FP3-QOD, FP3-ICD are represented in black, orange and light blue lines, respectively. **(C)**.

The root-mean-square deviation (RMSD) is often used to measure global conformational changes in macromolecular structures and has become an increasingly popular method for assessing the convergence of molecular simulations. The RMSDs of the backbone atoms of our systems were calculated with reference to the energy-minimized structure, which is very close to the crystal structures. Although the systems were relatively stable even before the first 200 ns of the simulation, we observed some distortions around 500 ns simulation time, for example, in the FP-2-ICD complex (Figure 3A). This indicated that the system has not fully stabilized even at 500 ns, and therefore, those time frames should not be considered for analyses. In comparison, all systems with FP3 were remarkably quite stable throughout the simulation time, suggesting less dynamic interaction of FP3 with the ligands. Nevertheless, we deemed the first 500 ns as an extension of equilibration and only considered the last 500 ns for further structural analyses. The local protein flexibility can be followed using per-residue root-mean-square fluctuation (RMSF) and can especially be informative in describing ligand-induced flexibility. From Figure 3B, we can observe dramatic fluctuations around residues 107–120, 222–226 in the FP2-QOD system and residues 59–62, 79–83 in the FP-2-ICD complex, compared to the apo FP-2. These residues constitute part of the α4/β2, β6-β7, α2-α3, α3/α4 loops (respectively) in FP-2 (Hogg et al., 2006), and therefore fluctuations are expected. Other observed regions of flexibility include residue 178 to 183 in FP2-QOD, which constitute a helical part of the protein. Therefore, the ligand, through its interaction with the protein, could also contribute to the more pronounced fluctuations by influencing the global fluctuation of the system. Similarly, a higher fluctuation of the loops formed by residues 88–91, 105–130, and 232–242 (belonging to α3/α4 and β6-β7 loops) was observed in the FP-3-ICD complex, which could be due to the same aforementioned reason (Figure 3C). On the contrary, the RMSF profile of the FP3-QOD system correlates very well with apo-FP3 RMSF, suggesting that QOD may not have a significant effect on the local flexibility of the protein.

### 3.2 QOD and ICD occupy the same pocket in the falcipain-2 active site

**FIGURE 4:**
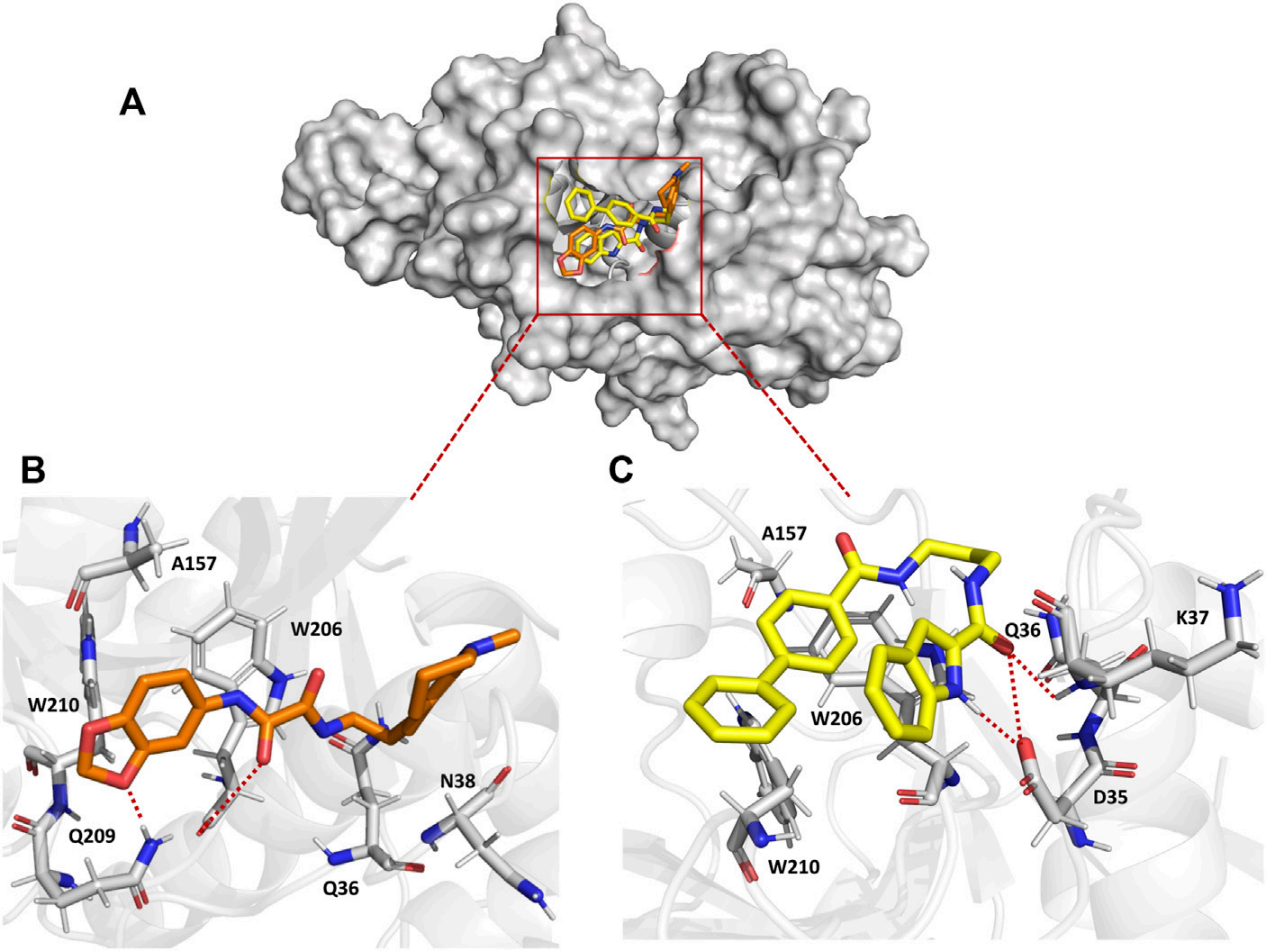
Surface representation of QOD (orange) and ICD (yellow) bound to the active site of FP-2 (gray) **(A)**. Interaction of QOD **(B)** and ICD **(C)** with the active site residues of FP-2. Hydrogen bonds are represented by red dotted lines.

To understand the binding mechanism of the ligands to FP-2, RMSD-based clustering was applied to the generated trajectories to classify the different conformations visited during the simulation. The classification produced 278 and 188 clusters for FP2-QOD and FP-2-ICD, respectively. In each case, the largest clusters (31.13% and 41.96% of the total frames for FP2-QOD and FP2-ICD) happened to be the only cluster in which the ligand is bound to the target. Interestingly, both ligands occupy the same binding pocket at the active site of FP-2 (Figure 4A) and are coordinated by almost identical FP-2 residues (Figures 4B, C). QOD interacts with Q36, N38, A157, W206, Q209, and W210, with hydrogen bonds formed by Q209 and W206 with one of the oxygen atoms of the dioxanyl ring and the carbonyl oxygen of the ethanediamide ligand backbone, being the major stabilizing interactions (Figure 4B). Similarly, ICD interacts with Q36, A157, W206, and W210, in addition to D35 and K37, both of which are responsible for hydrogen bonding with the formamide carbonyl oxygen of the ligand. An additional hydrogen bond is formed between the side chain carboxyl oxygen of D35 and the nitrogen atom of the indoyl ring of the ligand (Figure 4C). FP-2 is a member of the papain-like C1 cysteine proteases family that is characterized by catalytic quads of cysteine, histidine and asparagine, and glutamine (Martynov et al., 2015), represented by Q36, C42, H174 and N204 in FP-2 (Ettari et al., 2010). Our predicted interaction models suggest that access to these catalytic residues by the natural substrate is prevented by the ligands.

### 3.3 QOD and ICD interact with critical active site residue of FP-3

**FIGURE 5:**
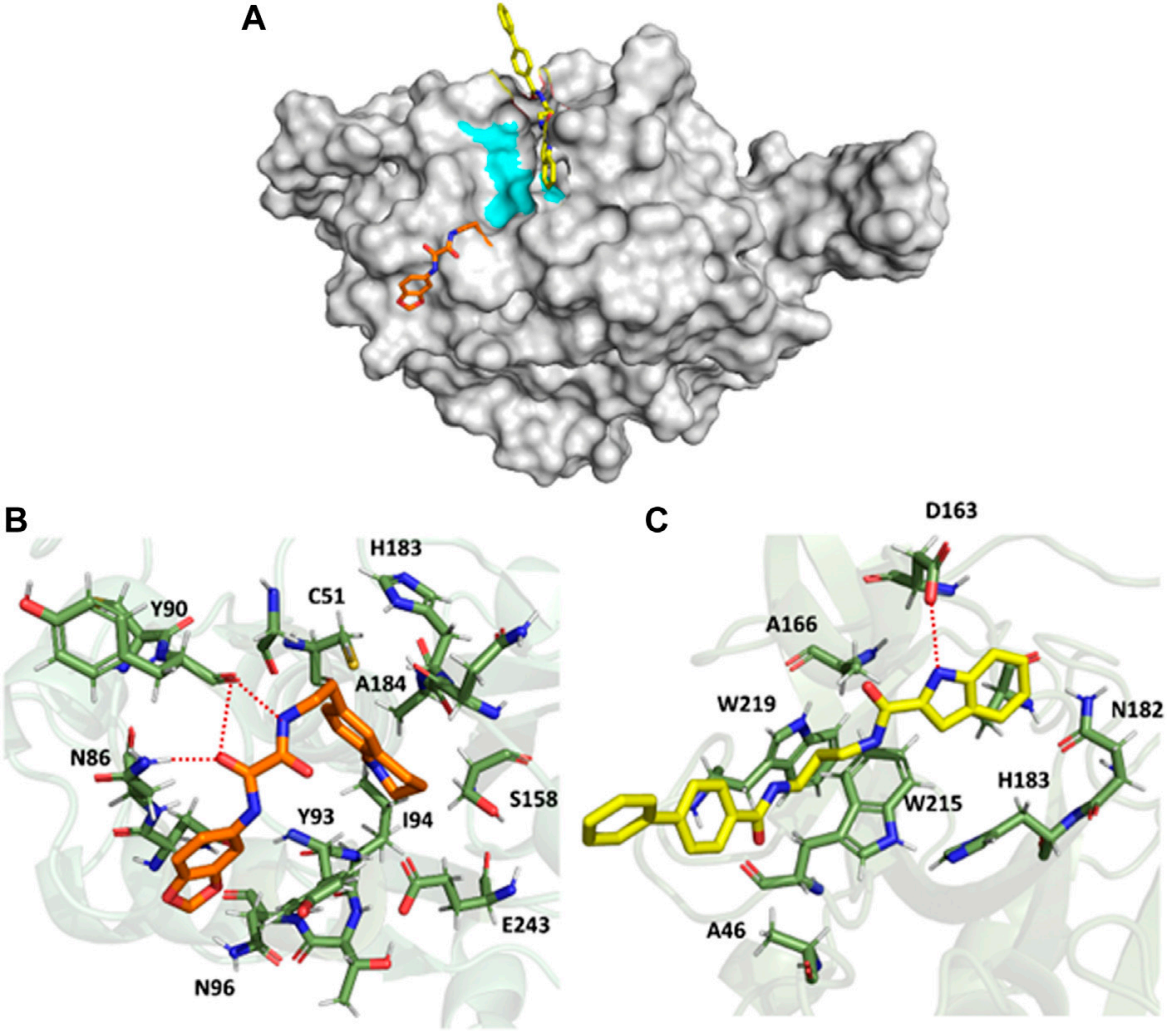
Surface representation of QOD (orange) and ICD (yellow) bound near the active site of FP-3 (gray). The critical catalytic residues are highlighted in cyan **(A)**. FP-3 residues (green) interacting with QOD **(B)** and ICD **(C)**. Hydrogen bonds are represented by red dotted lines.

Similarly, the trajectories from the simulation of the FP3-QOD and FP3-ICD complexes were clustered to classify the different conformations sampled. Just like in FP-2, the largest clusters (67.54% and 26.62% of the total frames for FP3-QOD and FP3-ICD, respectively) happened to be the only clusters where both ligands are bound to FP-3. Unlike in FP-2, the ligands are not fully embedded in the FP-3 active site and do not assume the same binding conformation but are docked very close to the critical residues, with the indonyl ring of ICD directly blocking the catalytic N213 from the top (Figure 5A). The interaction of FP-3 with QOD is maintained by a hydrogen bond between the side chain nitrogen atom of N86 and amide-oxygen of the ethanediamide backbone, as well as two hydrogen bonds formed between the backbone oxygen atom of Y90 and both N′-nitrogen and N-oxygen atom of the ligand ethanediamide backbone (Figure 5B). Other FP-3 residues that coordinate QOD include catalytic C51 and H183, Y93, I94, N96, S158, A184, and E243. The catalytic residues of the protein are not directly blocked in this interaction mode. However, the ligand’s methyl-tetrahydroquinolinyl ring is flexible and could move back and forth or even sideways to cause steric hindrance and prevent access to C51 and H183 for enzyme catalysis.

On the other hand, ICD forms only a single hydrogen bond with the side chain oxygen atom of D163 and the nitrogen atom of the ligand indonyl substituent. The amino acids A46, A166, N182, catalytic H183, W215, and W219 constituted the remaining residues interacting with ICD (Figure 5C). Notably, the ligand’s biphenyl rings appeared not to contribute significantly to this interaction, given that the rings protruded outside and were almost completely excluded from the protein surface (Figures 5A, C).

### 3.4 The predicted binding energies are consistent with the predicted interactions, and the experimental results

The calculated binding energies of QOD and ICD against FP-2 returned comparable values (Table 1). This is not surprising, given that both ligands occupy the same binding pocket at the receptor’s active site and are coordinated by almost identical residues (Figure 4). This is also consistent with the experimental IC_50_ values, which are 14.71 μM and 12.16 μM against FP-2, for QOD and ICD, respectively (Rana et al., 2020). The difference in the predicted binding energies of the ligands against FP-3 is surprisingly large (Table 1), considering their comparable IC50 values of 9.98 μM and 8.56 μM for QOD and ICD, respectively. However, this observation agrees, to some extent, with the predicted interactions since QOD exhibited more robust interaction with FP-3 in terms of the number of hydrogen bonds and the coordinating residues (Figure 5).

**TABLE 1:** MM/GBSA binding energy calculations.

Ligand	Predicted binding energy (kcal/mol)	
	FP-2	FP-3
QOD	−18.59 ± 3.67	−32.81 ± 3.68
ICD	−21.36 ± 2.65	0.00 ± 2.67

NB: Binding energies are reported as mean ± standard deviation. A detailed description of the contribution of the individual components of Eq. 1 to the total binding energies can be found in the supplementary datasheet.

## 4 Discussion

Continuous efforts towards the design of new and improvement of the current antimalarial drugs are necessary to address the current demand for managing antimalarial drug resistance (Komatsuya et al., 2013; Suzuki et al., 2015) as well as meeting the WHO goal of eradicating malaria by 2030 in at least 35 countries (WHO, 2022). In line with this, we probed recently reported dual inhibitors of *P. falciparum* important cysteine proteases (FP-2 and FP-3), QOD, and ICD (Rana et al., 2020), using molecular dynamics simulations to unravel the mechanism of their inhibitions of these enzymes. We allowed the ligands to diffuse freely in the simulation box in an attempt to enable unbiased sampling of the ligands’ preferred binding modes. After clustering the generated trajectories, we observed that both ligands preferred only single binding conformation, and those conformations represented the largest clusters sampled in all complexes. The ligands were unbound to the targets in all other clusters.

The predicted interaction model reveals that both compounds, QOD and ICD, docked to the same pocket in the FP-2 active site and interacted with the active site residues in an almost identical fashion. This observation is not surprising considering that these ligands were selected from a screening using a pharmacophore model of the active site inhibitors of FP-3 (Kerr et al., 2009b; 2009a; Rana et al., 2020), that FP-2 and FP-3 share 68% identity and also the conservation of catalytic residues in the two enzymes (Pandey and Dixit, 2012). The comparable calculated binding energies of the ligands (−18.59 ± 3.67 and −21.36 ± 2.65 kcal/mol for QOD and ICD, respectively) against FP-2 corroborated with their experimental IC_50_ values (Rana et al., 2020) and also the interaction mode.

Conversely, QOD and ICD assumed different yet closely positioned docking poses very near to FP-3’s catalytic residues. Compound QOD interacts with FP-3 more tightly than compound ICD, interacting with more residues, forming more hydrogen bonds, and the ligand fully embedded on the protein surface, compared to the latter, whose biphenyl substituent is wholly excluded from the interaction site and protein surface. In addition, the difference in their predicted binding energies was notably large, supporting stronger binding of QOD but incoherent with their experimental IC_50_ values (Rana et al., 2020). Notwithstanding, the QOD-FP-3 interaction model constitutes 67.54% of the total analyzed snapshots as opposed to the 26.62% for the ICD-FP-3 model, further pointing towards the more sustained and, therefore, stronger binding of QOD.

In conclusion, we have profiled QOD and ICD as active site inhibitors of FP-2 and FP-3. These compounds inhibit the activity of the FPs, most likely by preventing access to important catalytic residues in the enzymes’ active sites. Inhibition of FP-2 and FP-3 is characterized by compromised amino acid metabolism in *P. falciparum* (Hanspal et al., 2002). Capitalizing on this, several ongoing research programs are currently trying to design both peptidyl and small molecule inhibitors of FPs as potential antimalarial drugs (Chakka et al., 2015; Previti et al., 2017; Himangini et al., 2018; Hernandez Gonzalez et al., 2022). However, most of these projects are still in the early stages of drug development. Therefore, we present our work as a framework for optimizing these lead compounds and hope that it will stimulate more efforts toward discovering potent antimalarial drugs. Owing to the potential globalization of vector-borne diseases due to climate change (Balogun et al., 2016), the search for new drug candidates must remain continuous.

## Data Availability

The original contributions presented in the study are included in the article/Supplementary Materials; further inquiries can be directed to the corresponding author.
