# Supplementary material for: Microsecond-long simulation reveals the molecular mechanism for the dual inhibition of falcipain-2 and falcipain-3 by antimalarial lead compounds
Source: Front Mol Biosci. 2022 Dec 19;9:1070080. doi: 10.3389/fmolb.2022.1070080 (PMC9806354; doi:10.3389/fmolb.2022.1070080)
Supplement: Supplementary file 1 [file Table2.DOCX]

Supplementary Material

# Supplementary Data

**PARAMETERS FOR ENERGY MINIMIZATION**

; LINES STARTING WITH ';' ARE COMMENTS

; Parameters describing what to do, when to stop and what to save

integrator = steep ; Algorithm (steep = steepest descent minimization)

emtol = 1000.0 ; Stop minimization when the maximumforce < 10.0 kJ/mol

emstep = 0.01 ; Energy step size

nsteps = 50000 ; Maximum number of (minimization) steps to perform

; Parameters describing how to find the neighbors of each atom and

how to calculate the interactions

nstlist = 1 ; Frequency to update theneighbor list and long range forces

cutoff-scheme = Verlet

ns_type = grid ; Method to determine neighbor list (simple, grid)

rlist = 1.2 ; Cut-off for making neighbor list (short range forces)

coulombtype = PME ; Treatment of long range electrostatic interactions

rcoulomb = 1.2 ; long range electrostatic cut-off

vdwtype = cutoff

vdw-modifier = force-switch

rvdw-switch = 1.0

rvdw = 1.2 ; long range Van der Waals cut-off

pbc = xyz ; Periodic Boundary Conditions

DispCorr =no

**PARAMETERS FOR NVT ENSEMBLE EQUILIBRATION**

; Run parameters

integrator = md ; leap-frog integrator

nsteps = 50000 ; 2 * 50000 = 100 ps

dt = 0.002 ; 2 fs

; Output control

nstenergy = 500 ; save energies every 1.0 ps

nstlog = 500 ; update log file every 1.0 ps

nstxout-compressed = 500 ; save coordinates every 1.0 ps

; Bond parameters

continuation = no ; first dynamics run

constraint_algorithm = lincs ; holonomic constraints

constraints = h-bonds ; bonds to H are constrained

lincs_iter = 1 ; accuracy of LINCS

lincs_order = 4 ; also related to accuracy

; Neighbor searching and vdW

cutoff-scheme = Verlet

ns_type = grid ; search neighboring grid cells

nstlist = 20 ; largely irrelevant with Verlet

rlist = 1.2

vdwtype = cutoff

vdw-modifier = force-switch

rvdw-switch = 1.0

rvdw = 1.2 ; short-range van der Waals cutoff (in nm)

; Electrostatics

coulombtype = PME ; Particle Mesh Ewald for long-range electrostatics

rcoulomb = 1.2 ; short-range electrostatic cutoff (in nm)

pme_order = 4 ; cubic interpolation

fourierspacing = 0.16 ; grid spacing for FFT

; Temperature coupling

tcoupl = V-rescale ; modified Berendsen thermostat

tc-grps = Protein_L12 Water_and_ions ; two coupling groups - more accurate

tau_t = 0.1 0.1 ; time constant, in ps

ref_t = 298 298 ; reference temperature, one for each group, in K

; Pressure coupling

pcoupl = no ; no pressure coupling in NVT

; Periodic boundary conditions

pbc = xyz ; 3-D PBC

; Dispersion correction is not used for proteins with the C36 additive FF

DispCorr = no

; Velocity generation

gen_vel = yes ; assign velocities from Maxwell distribution

gen_temp = 298 ; temperature for Maxwell distribution

gen_seed = -1 ; generate a random seed

**PARAMETERS FOR NPT ENSEMBLE EQUILIBRATION**

; Run parameters

integrator = md ; leap-frog integrator

nsteps = 1000000 ; 2 ns

dt = 0.002 ; 2 fs

; Output control

nstxout = 500 ; save coordinates every 1.0 ps

nstvout = 500 ; save velocities every 1.0 ps

nstenergy = 500 ; save energies every 1.0 ps

nstlog = 500 ; update log file every 1.0 ps

; Bond parameters

continuation = yes ; Restarting after NVT

constraint_algorithm = lincs ; holonomic constraints

constraints = h-bonds ; bonds involving H are constrained

lincs_iter = 1 ; accuracy of LINCS

lincs_order = 4 ; also related to accuracy

; Nonbonded settings

cutoff-scheme = Verlet ; Buffered neighbor searching

ns_type = grid ; search neighboring grid cells

nstlist = 10 ; 20 fs, largely irrelevant with Verlet scheme

rcoulomb = 1.0 ; short-range electrostatic cutoff (in nm)

rvdw = 1.0 ; short-range van der Waals cutoff (in nm)

DispCorr = EnerPres ; account for cut-off vdW scheme

; Electrostatics

coulombtype = PME ; Particle Mesh Ewald for long-range electrostatics

pme_order = 4 ; cubic interpolation

fourierspacing = 0.16 ; grid spacing for FFT

; Temperature coupling is on

tcoupl = V-rescale ; modified Berendsen thermostat

tc-grps = Protein_L12 Water_and_ions ; two coupling groups - more accurate

tau_t = 0.1 0.1 ; time constant, in ps

ref_t = 298 298 ; reference temperature, one for each group, in K

; Pressure coupling is on

pcoupl = Parrinello-Rahman ; Pressure coupling on in NPT

pcoupltype = isotropic ; uniform scaling of box vectors

tau_p = 2.0 ; time constant, in ps

ref_p = 1.0 ; reference pressure, in bar

compressibility = 4.5e-5 ; isothermal compressibility of water, bar^-1

refcoord_scaling = com

; Periodic boundary conditions

pbc = xyz ; 3-D PBC

; Velocity generation

gen_vel = no ; Velocity generation is off

**PARAMETERS FOR PRODUCTION MD RUN**

; Run parameters

integrator = md ; leap-frog integrator

nsteps = 500000000 ; 2 * 250000000 = 1000000 ps (1us)

dt = 0.002 ; 2 fs

; Output control

nstxout = 0 ; suppress bulky .trr file by specifying

nstvout = 0 ; 0 for output frequency of nstxout,

nstfout = 0 ; nstvout, and nstfout

nstenergy = 5000 ; save energies every 10.0 ps

nstlog = 5000 ; update log file every 10.0 ps

nstxout-compressed = 5000 ; save compressed coordinates every 10.0 ps

compressed-x-grps = System ; save the whole system

; Bond parameters

continuation = yes ; Restarting after NPT

constraint_algorithm = lincs ; holonomic constraints

constraints = h-bonds ; bonds involving H are constrained

lincs_iter = 1 ; accuracy of LINCS

lincs_order = 4 ; also related to accuracy

; Neighborsearching

cutoff-scheme = Verlet ; Buffered neighbor searching

ns_type = grid ; search neighboring grid cells

nstlist = 10 ; 20 fs, largely irrelevant with Verlet scheme

rcoulomb = 1.0 ; short-range electrostatic cutoff (in nm)

rvdw = 1.0 ; short-range van der Waals cutoff (in nm)

; Electrostatics

coulombtype = PME ; Particle Mesh Ewald for long-range electrostatics

pme_order = 4 ; cubic interpolation

fourierspacing = 0.16 ; grid spacing for FFT

; Temperature coupling is on

tcoupl = V-rescale ; modified Berendsen thermostat

tc-grps = Protein_L12 Water_and_ions ; two coupling groups - more accurate

tau_t = 0.1 0.1 ; time constant, in ps

ref_t = 298 298 ; reference temperature, one for each group, in K

; Pressure coupling is on

pcoupl = Parrinello-Rahman ; Pressure coupling on in NPT

pcoupltype = isotropic ; uniform scaling of box vectors

tau_p = 2.0 ; time constant, in ps

ref_p = 1.0 ; reference pressure, in bar

compressibility = 4.5e-5 ; isothermal compressibility of water, bar^-1

; Periodic boundary conditions

pbc = xyz ; 3-D PBC

; Dispersion correction

DispCorr = EnerPres ; account for cut-off vdW scheme

; Velocity generation

gen_vel = no ; Velocity generation is off

**PARAMTERS FOR RUNNING MMGBSA FREE ENERGY CALCULATION**

# General namelist variables

&general

sys_name = "" # System name

startframe = 1 # First frame to analyze

endframe = 50001 # Last frame to analyze

interval = 1 # Number of frames between adjacent frames analyzed

forcefields = "oldff/leaprc.ff99SB,leaprc.gaff" # Define the force field to build the Amber topology

ions_parameters = 1 # Define ions parameters to build the Amber topology

PBRadii = 3 # Define PBRadii to build amber topology from GROMACS files

temperature = 298.15 # Temperature

qh_entropy = 0 # Do quasi-harmonic calculation

interaction_entropy = 0 # Do Interaction Entropy calculation

ie_segment = 25 # Trajectory segment to calculate interaction entropy

c2_entropy = 0 # Do C2 Entropy calculation

assign_chainID = 0 # Assign chains ID

exp_ki = 0.0 # Experimental Ki in nM

full_traj = 0 # Print a full traj. AND the thread trajectories

gmx_path = "" # Force to use this path to get GROMACS executable

keep_files = 2 # How many files to keep after successful completion

netcdf = 0 # Use NetCDF intermediate trajectories

solvated_trajectory = 1 # Define if it is necessary to cleanup the trajectories

verbose = 1 # How many energy terms to print in the final output

/

# (AMBER) Generalized-Born namelist variables

&gb

igb = 5 # GB model to use

intdiel = 1.0 # Internal dielectric constant for sander

extdiel = 78.5 # External dielectric constant for sander

saltcon = 0.0 # Salt concentration (M)

surften = 0.0072 # Surface tension

surfoff = 0.0 # Surface tension offset

molsurf = 0 # Use Connelly surface ('molsurf' program)

msoffset = 0.0 # Offset for molsurf calculation

probe = 1.4 # Solvent probe radius for surface area calc

ifqnt = 0 # Use QM on part of the system

qm_theory = "" # Semi-empirical QM theory to use

qm_residues = "" # Residues to treat with QM

qmcharge_com = 0 # Charge of QM region in complex

qmcharge_lig = 0 # Charge of QM region in ligand

qmcharge_rec = 0 # Charge of QM region in receptor

qmcut = 9999.0 # Cutoff in the QM region

scfconv = 1e-08 # Convergence criteria for the SCF calculation, in kcal/mol

peptide_corr = 0 # Apply MM correction to peptide linkages

writepdb = 1 # Write a PDB file of the selected QM region

verbosity = 0 # Controls the verbosity of QM/MM related output

alpb = 0 # Use Analytical Linearized Poisson-Boltzmann (ALPB)

arad_method = 1 # Selected method to estimate the effective electrostatic size

/
